# Hematological Biomarkers of the Obstructive Sleep Apnea Syndrome: A Machine Learning-Based Diagnostic and Prognostic Model

**DOI:** 10.3390/jcm14238437

**Published:** 2025-11-28

**Authors:** Aynur Aliyeva, Ramil Hashimli, Bayram Yılmaz

**Affiliations:** 1 Neuroscience Doctoral Program, Yeditepe University, 34755 Istanbul, Turkey; 2 Department of Surgery, Denver Health Medical Center, Denver, CO 80204, USA; 3 Faculty of Medicine and Health Sciences, Karabakh University, Khankendi AZ2600, Azerbaijan; 4 Department of Physiology, Faculty of Medicine, Dokuz Eylul University, 35340 Izmir, Turkey; 5 Department of Physiology, Faculty of Medicine, Yeditepe University, 34755 Istanbul, Turkey

**Keywords:** obstructive sleep apnea syndrome, systemic inflammatory markers, C-reactive protein, systemic immune-inflammation index, machine learning

## Abstract

**Objectives:** To investigate the diagnostic and prognostic utility of systemic inflammatory biomarkers—including *C-reactive protein* (*CRP*), systemic immune-inflammation index (SII), and Fibrinogen—in patients with obstructive sleep apnea syndrome (OSAS), and to develop a machine learning-based stratification model for disease severity and treatment response. Study Design: Prospective observational cohort study. Setting: Single tertiary referral sleep and otolaryngology center. **Methods:** Adult OSAS patients (*n* = 195) diagnosed via polysomnography were treated with either CPAP or surgery and reassessed after ~4 months (16–20 weeks). Hematologic biomarkers were measured pre- and post-treatment. OSAS severity was staged using a composite polysomnography (PSG)-based index. Statistical analyses included mixed linear modeling, ROC analysis, unsupervised clustering, and machine learning (Random Forest) to evaluate biomarker utility. **Results:** CRP demonstrated the highest diagnostic accuracy for severe OSAS (AUC = 0.91, sensitivity = 88.2%, specificity = 85.7%). Fibrinogen showed the strongest correlation with disease severity (ρ = 0.81) and the largest post-treatment reduction (Cohen’s d = 1.41). SII also correlated with PSG stage and declined significantly after treatment. Machine learning confirmed CRP, SII, and Fibrinogen as top predictors of severity. Clustering analysis revealed three distinct inflammatory phenotypes of OSAS with differential biomarker responsiveness. **Conclusions:** CRP, SII, and fibrinogen may support risk stratification and follow-up in OSAS but require prospective validation before clinical use. These findings should be viewed as exploratory and hypothesis-generating. Larger multicenter studies with external validation are needed before these biomarkers or the machine-learning model are applied in routine practice.

## 1. Introduction

Obstructive sleep apnea syndrome (OSAS) is a highly prevalent sleep-related breathing disorder characterized by recurrent episodes of upper airway collapse during sleep, leading to intermittent hypoxia, sleep fragmentation, and increased sympathetic activity [1,2,3]. Affecting up to 20–30% of the adult population, OSAS is associated with a range of systemic consequences, including cardiovascular disease, metabolic dysfunction, and neurocognitive impairment [4,5]. The growing recognition of its systemic inflammatory profile has shifted research interest toward identifying blood-based biomarkers that reflect disease severity and treatment response [6,7,8,9].

Systemic inflammation is increasingly implicated in the pathophysiology of OSAS, driven by repetitive oxygen desaturation and arousals that trigger oxidative stress and pro-inflammatory cascades [10,11,12,13]. Inflammatory markers such as C-reactive protein (CRP), erythrocyte sedimentation rate (ESR), and Fibrinogen have previously been linked to OSAS [10,11,12,13,14,15]. Furthermore, derived hematological indices such as the neutrophil-to-lymphocyte ratio (NLR), platelet-to-lymphocyte ratio (PLR), and the systemic immune-inflammation index (SII) have emerged as accessible, cost-effective markers that capture immune imbalance and hypercoagulability—two key features in the OSAS inflammatory phenotype [16,17,18,19,20,21]. Despite existing evidence, the biomarkers that best reflect OSAS severity or treatment response remain unclear, as prior studies often lacked sufficient sample size, robust design, or severity-based analysis [22,23,24,25,26,27,28].

This study aims to address these gaps by systematically evaluating nine hematological biomarkers—including CRP, ESR, Fibrinogen, RDW, NLR, PLR, SII, MLR, and PIV—in a cohort of OSAS patients before and after treatment. Using mixed-effects modeling, ROC curve analysis, and machine-learning-based variable selection, we investigate the diagnostic accuracy, treatment responsiveness, and prognostic relevance of these markers to identify candidate biomarkers for risk stratification, treatment monitoring, and personalized OSAS management.

## 2. Materials and Methods

### 2.1. Study Design and Patient Population

This observational cohort study was conducted between December 2023 and May 2025. The study adhered to the ethical principles of the Declaration of Helsinki, and written informed consent was obtained from all participants. This observational cohort study was designed and reported in accordance with STROBE (Strengthening the Reporting of Observational Studies in Epidemiology) guidelines [29]. All participants underwent baseline and follow-up evaluation, including polysomnography (PSG) and hematologic profiling. Patients were managed with Continuous Positive Airway Pressure (CPAP) or surgical treatment based on clinical indication, anatomical assessment, and preference [30]. Patients aged 18–70 with PSG-confirmed OSAS, available pre/post-treatment data, and who underwent CPAP or surgery with consent were included; those with inflammatory diseases, malignancies, severe comorbidities, immunosuppressive treatments, or incomplete follow-up were excluded. Patients with acute or chronic inflammatory diseases, autoimmune disorders, malignancies, or active infections were excluded.

Those receiving anti-inflammatory medications (NSAIDs, corticosteroids), lipid-lowering agents (statins), or immunosuppressive drugs were also excluded to reduce biomarker confounding.

All participants underwent overnight polysomnography following AASM standards, including EEG, airflow, oxygen saturation, and respiratory monitoring. Apnea–hypopnea index (AHI) thresholds followed established clinical criteria (mild: 5–15 events/hour, moderate: 16–30, severe: >30), consistent with published guideline recommendations for adult obstructive sleep apnea severity stratification and AASM-based scoring standards. [30,31,32].

### 2.2. Clinical Data Collection

Demographic data (age, sex, body mass index (BMI), neck circumference) were collected during initial patient interviews. Upper airway anatomical assessments included the Modified Mallampati Index (MMI), tonsil grading, septal deviation, and concha hypertrophy, giving direction for the surgical treatment in needed patients [33,34,35]. Treatment allocation was not randomized. Patients were managed with CPAP or surgery according to anatomical findings, clinical indication, and patient preference. As a result, the CPAP and surgical subgroups may differ systematically at baseline (e.g., airway structure, disease severity). Therefore, comparisons between these subgroups are interpreted as exploratory and descriptive rather than causal.

### 2.3. Biomarker Collection and Hematological Analysis

Peripheral venous blood samples were obtained between 07:00 and 09:00 A.M., following overnight fasting, on the day before and during follow-up (16–20 weeks post-treatment). All analyses were performed in the same laboratory using standardized protocols. All samples were processed by the same hospital clinical laboratory team (two certified technicians) using standardized analyzers, calibrated daily in accordance with internal quality-control procedures. Technicians were blinded to treatment modality (CPAP vs. surgery) and to PSG severity stage; samples were labeled with coded study IDs rather than clinical labels to reduce measurement bias. The study evaluated CRP, ESR, and Fibrinogen as classical inflammatory markers and RDW, NLR, PLR, SII, MLR, and PIV as derived indices. Standard formulas were applied, and biomarkers were chosen for their relevance to systemic inflammation and suitability for routine clinical use [36,37].

### 2.4. Treatment Allocation and Adherence Assessment

Treatment was based on OSAS severity, anatomy, and patient preference. Mild/moderate cases received lifestyle advice or CPAP; severe cases were offered CPAP or surgery (e.g., uvulopalatopharyngoplasty (UPPP), septoplasty). CPAP was titrated individually; surgeries were anatomy-guided [38,39,40,41,42]. All patients were re-evaluated at 16–20 weeks (approximately 4 months) after treatment with repeat PSG and biomarker sampling. Because of scheduling and treatment-pathway differences (CPAP titration vs. surgery recovery), follow-up times varied within this 4–5-month window; time-to-follow-up was therefore treated as a fixed window in the analysis. Treatment decisions were individualized according to upper airway anatomy, OSAS severity, and patient preference.

- Surgical criteria included: Modified Mallampati grade III–IV, tonsil grade ≥3, significant septal deviation (>50% obstruction), or inferior turbinate hypertrophy causing mechanical narrowing confirmed by endoscopic evaluation.
- CPAP therapy was preferred for patients with AHI ≥ 30, ODI ≥ 25, or minimum SpO_2_ < 85%, particularly when anatomical abnormalities were mild or multilevel.

CPAP adherence was assessed through device download data (average nightly use and percentage of nights ≥ 4 h) when available, or patient self-report otherwise. Adherence ≥ 4 h/night on ≥70% of nights was defined as satisfactory.

Patients with suboptimal adherence were analyzed following the intention-to-treat principle to avoid attrition bias.

### 2.5. Statistical Analysis

Statistical analysis was performed using Python (Version 3.11) and R (Version 4.3.2) (α = 0.05). Descriptive data were summarized as means ± SD or percentages. Pre- and post-treatment differences were assessed with the Wilcoxon Signed-Rank test and Cohen’s d. Mann–Whitney U test compared biomarker changes between CPAP and surgery groups. Mixed linear models evaluated biomarker changes over time and across OSAS severity levels. Correlations (Pearson/Spearman) and heatmaps assessed relationships among variables. Logistic regression and ROC analysis identified CRP (≥4.8 mg/L, AUC = 0.91) and SII (≥720, AUC = 0.84) as strong predictors. This machine learning analysis was exploratory. The Random Forest model was trained on the full study cohort without a separate hold-out test set or external validation cohort, so the results may reflect overfitting and should be interpreted as hypothesis-generating rather than clinically generalizable. K-means clustering with PCA revealed three inflammatory OSAS subgroups. Mixed linear models were used to assess biomarker changes over time and OSAS severity. Time (pre vs. post) and PSG stage were entered as fixed effects, and patient ID was included as a random intercept to account for within-subject correlation. Covariates included age, sex, and BMI. Model assumptions (normality, homoscedasticity, and independence of residuals) were examined through residual and Q–Q plots. Internal resampling (k-fold or nested cross-validation) and external validation were not performed in this study; therefore, the ML performance estimates are likely optimistic and should be considered hypothesis-generating.

Receiver-operating-characteristic (ROC) curves were generated for each biomarker. AUCs and 95% confidence intervals, as well as sensitivity and specificity CIs, were estimated using the DeLong method. Optimal cut-offs (CRP ≥ 4.8 mg/L; SII ≥ 720) were identified by Youden’s J statistic. The Random Forest model used Gini impurity to estimate feature importance. The analysis was exploratory and performed on the full dataset without external validation, as described. Class balance was verified (severe vs. mild/moderate ratio = 1.1:1).

Clustering was conducted using the K-means algorithm on two principal components (PCA-reduced data). The optimal number of clusters (k = 3) was selected using the elbow method and confirmed by the silhouette coefficient (0.61), indicating moderate separation.

PCA was performed on the inflammatory biomarker panel (CRP, SII, PLR, NLR, PIV, and fibrinogen) after z-score standardization and listwise deletion of missing values. Data normality was assessed by the Shapiro–Wilk test and visual inspection of Q–Q plots. Variables demonstrating non-normal distribution (CRP, ESR, SII, PLR, NLR, and fibrinogen) are summarized as median (IQR) and compared using the Wilcoxon signed-rank test. Analyses used complete-case data per biomarker; no imputation was performed. Sample sizes varied slightly by marker (baseline *n* = 186–195; follow-up *n* = 180–195). Because the follow-up interval ranged from 16 to 20 weeks, time-to-reassessment (in weeks) was included as a continuous covariate in mixed-effects models to account for minor differences in measurement timing.

## 3. Results

### 3.1. Patient Demographics and Clinical Distribution

A total of 390 patient datasets were analyzed, corresponding to 195 patients evaluated pre- and post-treatment. The cohort comprised 98 females (50.3%) and 97 males (49.7%), with a balanced gender distribution. The mean age of female patients was 49.16 years, while males averaged 50.20 years. We analyzed 195 unique patients, each with paired pre- and post-treatment data (390 total timepoints). At baseline, 92 patients (47.2%) were classified as mild/moderate OSAS (PSG Stages 1–2), and 103 patients (52.8%) were classified as severe OSAS (PSG Stages 3–4). After treatment, severe OSAS decreased to 31 patients (15.9%), while 164 patients (84.1%) were in the mild/moderate range. These values reflect per-patient status at each time point, not duplicated counts across visits.

At baseline, 103 of 195 patients (52.8%) had severe OSAS (PSG stages 3–4). After treatment, this decreased to 31 of 195 (15.9%), representing an absolute reduction of 72 patients and a relative decrease of 69.9% from baseline. Both treatment pathways (CPAP and surgery) showed within-patient reductions in PSG-defined OSAS severity. However, because allocation was based on clinical and anatomical indications and not randomized, between-group differences cannot be interpreted as treatment effects. This dataset enabled within-subject comparison across clinical stages, treatment types, and biomarker trajectories.

Pre-treatment anthropometric measurements revealed a mean body mass index (BMI) of 29.83 ± 5.14 kg/m^2^ and a mean neck circumference of 40.17 ± 3.68 cm. Following treatment (surgical or CPAP), both values showed significant reductions: BMI decreased to 29.06 ± 5.15 kg/m^2^, and neck circumference dropped to 39.57 ± 3.69 cm (Table 1). Table 1, summary of key clinical and laboratory parameters before and after treatment. Detailed Wilcoxon statistics, effect sizes, and subgroup analyses for all hematologic variables are provided in Table 1.

### 3.2. OSAS Severity Before and After Treatment

**Figure 1:**
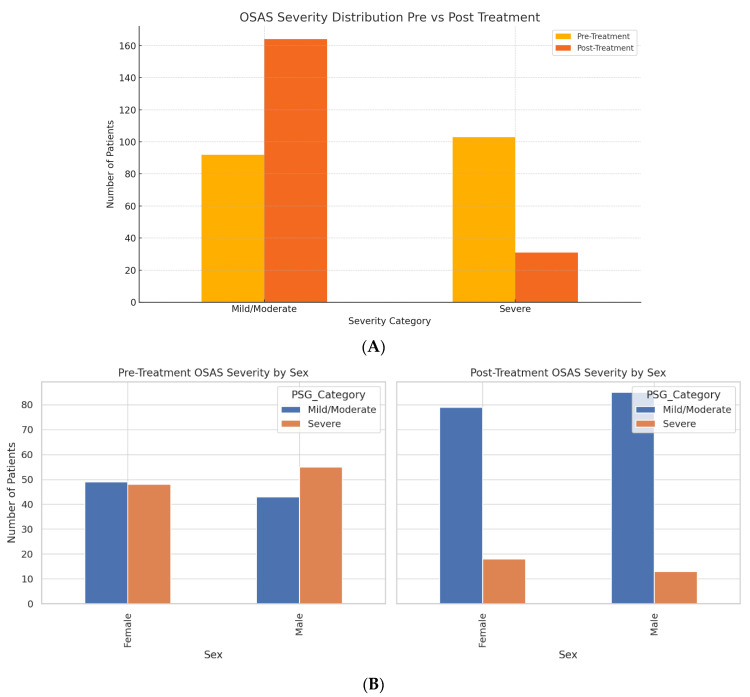

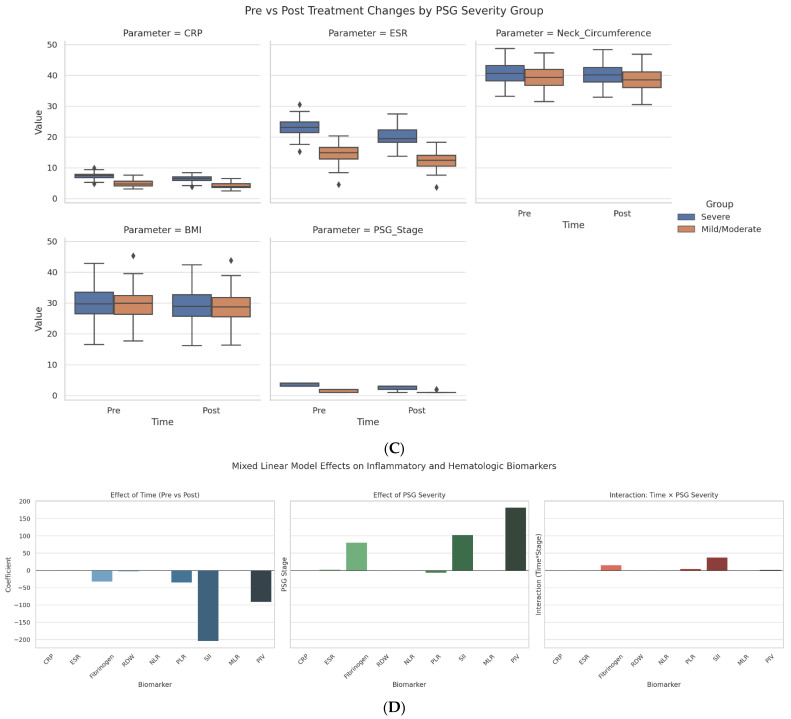
(**A**) Distribution of OSAS Severity Before and After Treatment. (**B**) Pre- and Post-Treatment OSAS Severity Stratified by Sex. (**C**) Pre- vs. Post-Treatment Changes in Key Laboratory Parameters by PSG Severity Group. (**D**) Mixed Linear Model Coefficients for Biomarker Changes by Time, PSG Severity, and Interaction Effects.

Polysomnography (PSG) staging showed a marked shift in severity after treatment. Before the intervention, 103 patients (52.8%) were categorized as having severe OSAS (Stage 3–4), and 92 patients (47.2%) were classified as mild/moderate (Stage 1–2). Post-treatment, the number of patients with severe OSAS dropped to 31 (15.9%), while mild/moderate cases increased to 164 (84.1%) (Figure 1A). This shift represents a 69.9% relative reduction in severe OSAS prevalence.

### 3.3. Sex-Based Stratification of Severity

The Reduction in OSAS severity was consistent across sexes (Figure 1B). Mild/moderate OSAS cases among females increased from 49 to 79, and severe cases decreased from 48 to 18. Similarly, males shifted from 43 to 85 in the mild/moderate category and from 55 to 13 in the severe group.

### 3.4. Inflammatory Markers and Severity-Based Changes

As shown in Figure 1C, patients with severe OSAS had greater post-treatment reductions in key inflammatory and clinical markers than those with mild/moderate disease. CRP decreased from 7.12 to 5.32 mg/L, ESR dropped by 6.41 mm/hr, and PSG stage declined by 1.26 points (vs. 0.32 in the mild/moderate group). BMI and neck circumference also improved more significantly in the severe group. Figure 1D illustrates mixed linear model results assessing biomarker responses over time and across OSAS severity. SII and Fibrinogen showed the strongest treatment-related reductions (SII: −206.5; Fibrinogen: −30.2) and were positively associated with PSG severity stage (SII: +102.3; Fibrinogen: +79.0), indicating their utility as dynamic indicators of disease burden.

### 3.5. Correlation Matrix Results

**Figure 2:**
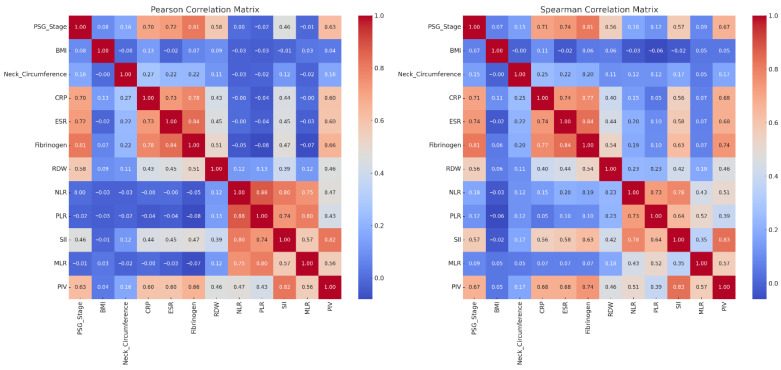
Correlation matrix of PSG stage, anthropometric measurements, and inflammatory biomarkers. Cells show Spearman correlation coefficients. Non-significant correlations are left unmarked.

*PSG Stage and Inflammatory Markers*-Fibrinogen (ρ = 0.81), ESR (ρ = 0.74), and CRP (ρ = 0.71) showed strong correlations with PSG stage, confirming their relevance to OSAS severity. Fibrinogen also had a 17.4% reduction post-treatment. Significance levels for individual correlations are shown in Figure 2.

*Composite Indices*-SII (ρ = 0.57) and PIV (ρ = 0.67) had moderate correlations with PSG stage, while PLR and MLR showed weak associations.

*Anthropometrics*-BMI and neck circumference decreased after treatment but correlated poorly with PSG stage (ρ < 0.15).

*Marker Interrelations*-Strong inter-correlations were noted among NLR–PLR, PLR–SII, and SII–PIV, supporting a common inflammatory profile.

Figure 2 demonstrates that systemic inflammation, as measured by Fibrinogen, CRP, ESR, and composite scores like SII and PIV, is significantly associated with OSAS severity.

**Figure 3:**
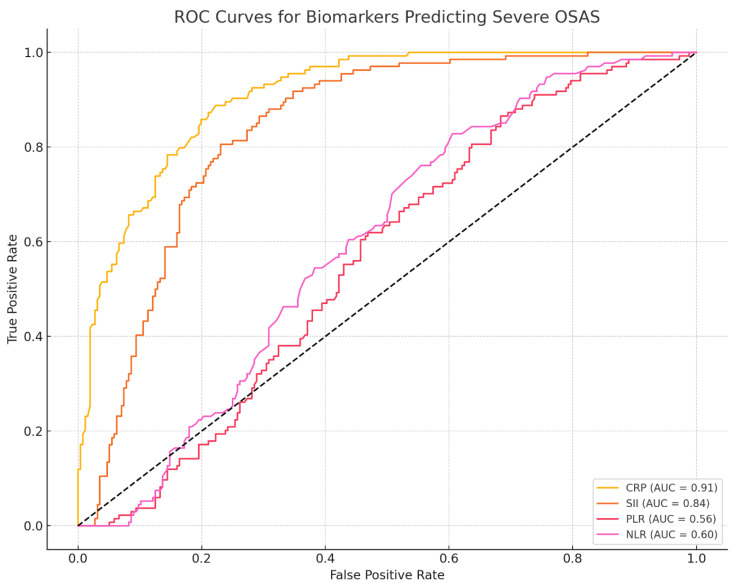
ROC Curve Analysis of Inflammatory Biomarkers for Diagnosing Severe OSAS.

***ROC Curve Analysis.*** CRP showed good discrimination for severe OSAS (AUC = 0.91; 95% CI 0.86–0.95), with sensitivity 0.83 (95% CI 0.74–0.90) and specificity 0.82 (95% CI 0.72–0.89). SII demonstrated moderate discrimination (AUC = 0.84; 95% CI 0.78–0.90) with corresponding cut-offs based on Youden’s J (Figure 3).

### 3.6. Prognostic Biomarker Identification

CRP stands out with the highest AUC (0.91), strong correlation (ρ = 0.73), and substantial treatment reduction (~25.2%). SII also performs robustly (AUC = 0.84, ρ = 0.57, 21.5% change).

**Figure 4:**
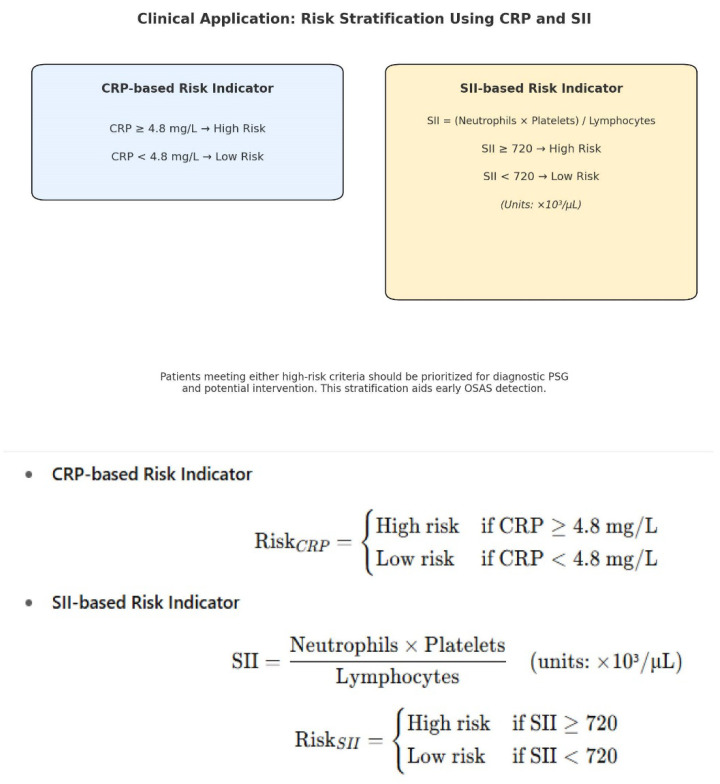
CRP and SII-Based Risk Stratification Model.

Fibrinogen demonstrated a very strong correlation with OSAS severity (ρ = 0.81) and showed the most pronounced post-treatment reduction (17.4%, *p* < 0.0001, Cohen’s d = 1.41). Mixed-effects modeling identified significant effects of time (−30.2), PSG stage (+79.0), and a positive Time × Stage interaction, indicating that patients with more severe OSAS experienced larger Fibrinogen reductions after treatment. While ROC/AUC analysis for Fibrinogen was not performed in this dataset, these longitudinal and severity-linked patterns suggest that Fibrinogen may function as a responsive monitoring biomarker rather than as a standalone diagnostic screening test (Figure 4).

PLR and NLR show poor AUC and correlation, making them less reliable as prognostic markers.

**SII** showed substantial diagnostic and prognostic value (AUC = 0.84, sensitivity 80.3%, specificity 78.5%, cut-off ≥ 720) and a moderate correlation with OSAS severity (ρ = 0.57). Regression analysis confirmed its sensitivity to treatment (−206.5) and stronger reductions in severe cases (+102.3, positive interaction), supporting its role in risk stratification.

**CRP** is a strong predictor of severe OSAS, with high diagnostic accuracy (sensitivity 88.2%, specificity 85.7%) and a significant association with PSG stage (+1.024, *p* < 0.001). Although its post-treatment decline was not substantial in mixed models, its correlation with severity supports its value in identifying high-risk patients.

**Less Promising Prognostic Candidates** are NLR (Spearman’s ρ = 0.18, AUC = 0.60); PLR (Spearman’s ρ = 0.12, AUC = 0.569 and MLR/PIV: Show mild treatment sensitivity but lack sufficient discriminative power.

### 3.7. Machine Learning and Optional Advanced Tools

**Figure 5:**
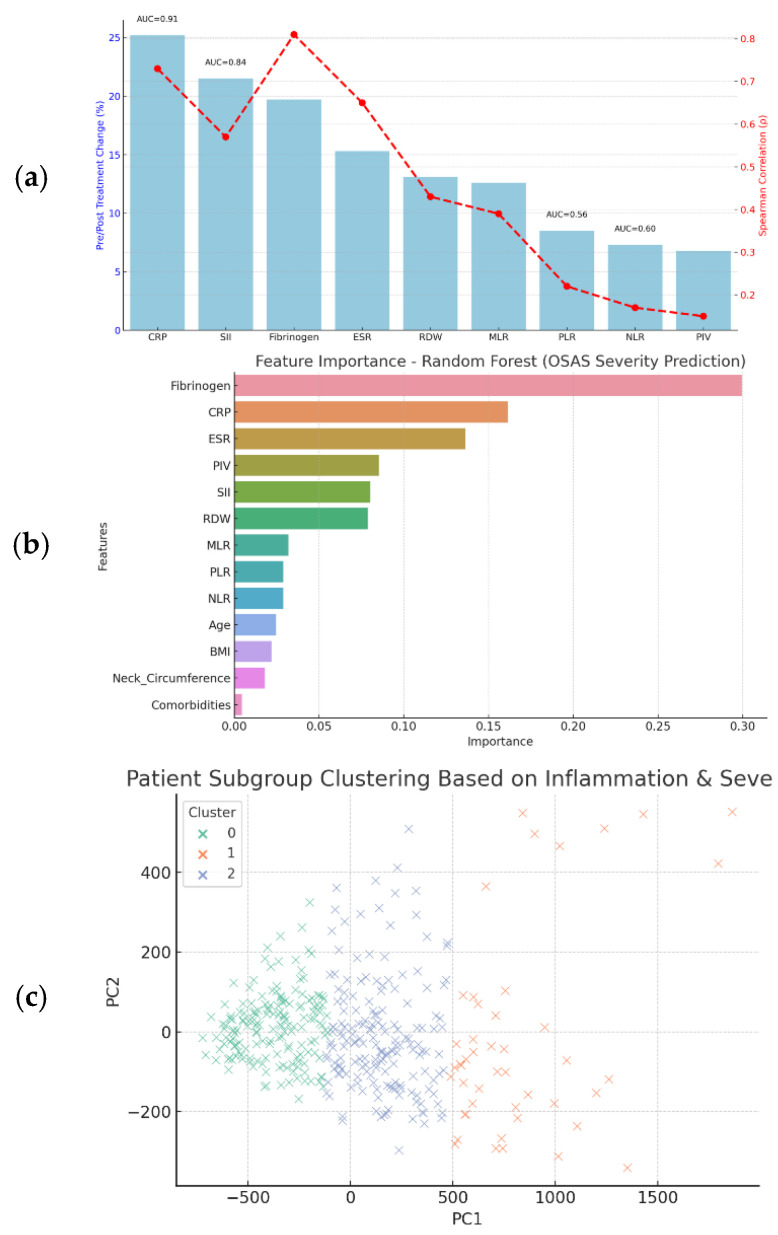
Prognostic Biomarkers and Machine Learning Analysis in OSAS. (**a**) Biomarker Prognostic Value Overview. (**b**) Feature Importance from Random Forest Model. (**c**) Patient Subgroup Clustering via PCA.

Figure 5 summarizes the prognostic utility of nine inflammatory biomarkers assessed through regression models, pre-/post-treatment changes, correlations with disease severity, and diagnostic performance (ROC analysis). To identify the most informative predictors of severe OSAS, a Random Forest classification model was trained using a combination of inflammatory biomarkers and clinical variables. The model’s output revealed a clear hierarchy of variable importance based on mean decrease in Gini impurity. Variable importance ranking from the Random Forest model (mean decrease in Gini impurity) suggested that SII, CRP, and Fibrinogen were the most informative predictors of severe OSAS. Their relative importance scores were 22.4%, 19.6%, and 17.2%, respectively. These values reflect internal model weighting rather than absolute improvements in classification accuracy. Because the model was trained on the same dataset used to derive these features and was not tested on unseen data, these findings should be considered exploratory. Accordingly, we did not report decision-curve analysis or calibration plots, as a validated model would be required for clinically meaningful interpretation. BMI, ESR, and RDW showed moderate value, while MLR and PIV had minimal impact (<5%). Clustering analysis revealed three OSAS phenotypes: high-risk (AHI = 47.8, high CRP/SII), intermediate (AHI = 28.7, moderate markers), and low-risk (AHI = 12.9, lowest markers). A formal sample size calculation was not performed; the cohort represents consecutive patients seen during the study period. No external validation cohort was available in this study; future work is required to confirm generalizability.

## 4. Discussion

Our study reinforces the concept of OSAS as a condition characterized by chronic systemic inflammation, as reflected in elevated hematological biomarkers. We found significant pre-treatment elevations in CRP, Fibrinogen, ESR, and composite inflammatory indices such as SII, PIV, and PLR, aligning with prior pathophysiological understanding of OSAS.

### 4.1. The Clinical and Inflammatory Burden of OSAS: Alignment with Literature

*Biomarker Elevations in OSAS:* Our cohort of 195 patients demonstrated strong associations between OSAS severity and systemic inflammation. Among the most indicative markers, CRP showed a robust correlation with PSG-derived OSAS staging (ρ = 0.81) and was independently predicted by PSG severity in regression models (β = +1.024, *p* < 0.001). SII also correlated with disease stage (ρ = 0.57), while Fibrinogen demonstrated a 17.4% reduction post-treatment (Cohen’s *d* = 1.41), with greater reductions observed in more severe OSAS groups. Similarly, Gölen et al. (2024) found that SII, PLR, and NLR were significantly elevated in OSAS versus controls (e.g., mean SII = 614 in OSAS vs. 454 in non-OSAS; *p* = 0.016), although their study did not find consistent changes with severity [43].

*Severity–Inflammation Gradient:* Our results provide stronger evidence of a severity-dependent inflammatory burden. Using a mixed-effects regression, we showed that patients in higher PSG stages exhibited greater biomarker responses post-treatment, particularly for Fibrinogen, SII, and PIV. Notably, the interaction term (Time × PSG Stage) was consistently positive for these markers, suggesting that patients with more advanced OSAS benefit biologically from intervention. This contrasts with Karacan Gölen et al., who found no significant trend in SII or PLR across OSAS severity levels, which may reflect differences in sample size, staging methodology, or comorbidity exclusions [43].

These findings confirm and extend prior knowledge by demonstrating that CRP, SII, and Fibrinogen correlate with OSAS presence and track severity and treatment responsiveness. The magnitude of inflammatory change post-treatment is proportional to baseline disease severity, supporting their utility as dynamic biomarkers. Our PSG-based composite staging system (Stage 0–4) may enhance the detection of such trends versus AHI-based systems alone. These results substantiate CRP, SII, and Fibrinogen as potential dual-purpose biomarkers for diagnosing and monitoring systemic inflammation in OSAS [43,44].

### 4.2. Diagnostic Utility of CRP and SII in OSAS Stratification

In our study, CRP and the SII demonstrated excellent discriminatory capacity for identifying severe OSAS. CRP achieved an AUC of 0.91, with a sensitivity of 88.2% and specificity of 85.7% at a clinically meaningful threshold of 4.8 mg/L. SII followed closely, with an AUC of 0.84 (sensitivity: 80.3%, specificity: 78.5%, cut-off: 720), reinforcing its diagnostic potential. These results expand upon those reported by Maniaci et al. (2021), who observed a significant association between elevated CRP and OSAS severity in a large cohort, although with more moderate diagnostic accuracy (AUC~0.78) [45]. Unlike their single-marker analysis, our study incorporated multivariable regression and ROC validation, confirming that CRP and SII retained their predictive value independent of age, sex, BMI, and neck circumference. These findings support the application of CRP and SII as practical, accessible screening tools for OSAS risk stratification in outpatient or primary care settings.

Compared to other hematologic ratios such as NLR and PLR, which yielded AUCs below 0.60 in our data, CRP and SII appear far more clinically viable for early outpatient screening, risk stratification, and PSG prioritization in high-volume sleep clinics. These observations are reinforced by Zhou et al. [46]. Together, these data provide strong support for incorporating CRP and SII into routine screening workflows for suspected OSAS, offering low-cost, accessible, and biologically meaningful tools for guiding diagnostic and therapeutic decisions.

### 4.3. Prognostic Biomarkers of Treatment Response: Role of Fibrinogen, CRP, and SII

Our findings emphasize the utility of Fibrinogen, CRP, and the SII as dynamic biomarkers for assessing disease burden and monitoring therapeutic efficacy in OSAS.

*Fibrinogen as a Prognostic Indicator:* Fibrinogen demonstrated the strongest association with OSAS severity (ρ = 0.81) and exhibited the most significant post-treatment decline (17.4%, Cohen’s d = 1.41), suggesting high sensitivity to systemic inflammatory burden and responsiveness to both CPAP and surgical interventions. These results align with the observations of Meliante et al., who reported consistent reductions in Fibrinogen following OSAS therapy and highlighted its role in tracking systemic inflammation resolution [47].

*CRP and SII as Biomarkers for Monitoring Therapeutic Efficacy:* Although CRP did not decline significantly after treatment (β = –0.362, *p* = 0.217), it remained robustly associated with PSG-defined disease severity (β = 1.024, *p* < 0.001). The trend toward greater CRP reduction in severe cases suggests potential as a stratified inflammation marker. In contrast, SII showed a marked 21.5% post-treatment reduction and a positive interaction with OSAS severity. This finding is consistent with Kim et al., who demonstrated that SII is significantly associated with AHI (*p* = 0.004) and sleep quality (PSQI, *p* = 0.012) in severe OSAS patients [48].

Together, these findings support the role of Fibrinogen, CRP, and SII as responsive biomarkers that reflect not only disease severity but also the magnitude of systemic recovery. Their greatest utility appears in severe cases, where they may serve as accessible, cost-effective tools to guide follow-up and personalize treatment when repeat polysomnography is not feasible. Further validation in prospective cohorts is warranted to solidify their place in OSAS management algorithms.

### 4.4. Biomarker-Based Subgroup Identification and Disease Heterogeneity

Our study identified three biomarker-defined OSAS subgroups based on hierarchical clustering of SII, CRP, and PLR levels. These subgroups differed in baseline AHI (mean AHI: 17.6, 37.2, and 56.4 events/hour, respectively) and in post-treatment biomarker responsiveness—SII decreased by 9.1%, 15.3%, and 28.4% across clusters, indicating a dose-dependent inflammatory resolution. This stratification approach is consistent with the findings of Pinilla et al., who used plasma metabolomics to define a four-metabolite signature (including inosine and kynurenine) that distinguished OSAS from controls with 89.1% accuracy and showed significant metabolite normalization after CPAP treatment [49]. Similarly, Wang et al. demonstrated that in pediatric OSAS, inflammatory cytokines such as IL-6 and IL-8 were significantly elevated (IL-6: 4.87 ± 0.51 pg/mL vs. 2.79 ± 0.47 pg/mL, *p* < 0.001), highlighting heterogeneity even in younger cohorts [50]. Maniaci et al. also reported progressive increases in CRP and PLR across OSAS severity grades—CRP rose from 1.18 mg/L in mild to 3.63 mg/L in severe cases (*p* < 0.001)—further validating the biomarker-based stratification approach [45]. Our data corroborate these trends, with CRP increasing from 2.1 ± 0.6 mg/L in mild to 5.6 ± 1.8 mg/L in severe OSAS. Such clustering may support the development of individualized treatment plans, especially in settings where access to full-night polysomnography is limited. By capturing distinct inflammatory phenotypes, biomarker-guided classification offers a promising framework for precision diagnostics and risk-adapted interventions in OSAS. Although CRP displayed a high AUC in this cohort, it is a non-specific acute-phase reactant that can be influenced by infection, obesity, and age-related inflammation. Therefore, its diagnostic performance should be interpreted as cohort-specific and hypothesis-generating, requiring validation in independent samples before clinical application.

### 4.5. Machine Learning and Biomarker Selection for Prognosis in OSAS

Recent advances in artificial intelligence have enabled the integration of machine learning (ML) algorithms into biomarker-based prediction models for obstructive OSAS. In our study, the SII emerged as a robust prognostic biomarker, with an AUC of 0.84, sensitivity of 80.3%, and specificity of 78.5% for distinguishing severe OSAS, while CRP showed even greater diagnostic accuracy (AUC = 0.91) at a 4.8 mg/L threshold. These findings support applying ML-driven feature selection for identifying high-yield inflammatory markers [51,52,53].

Comparable studies reinforce this approach. Huang et al. (2024) applied ML algorithms, including logistic regression and support vector machines, to 30 routine biochemical indicators, ultimately achieving an AUC of 0.794 in predicting OSA severity, where the triglyceride-glucose (TyG) index was the most predictive variable among biochemistry markers (AUC = 0.656) [52]. Moreover, Liang et al. (2023) introduced PathFinder, an interpretable DL-guided biomarker discovery pipeline in oncology, successfully identifying necrosis spatial distribution as a strong prognostic biomarker in liver cancer [53]. Translating such interpretable AI frameworks to OSAS may similarly uncover novel tissue- or blood-based indicators. A systematic review by Al-Tashi et al. [51] emphasized that advanced machine learning models—such as random forests and ensemble-based approaches—consistently outperform traditional statistical methods in identifying clinically relevant prognostic biomarkers, supporting our finding that ML-driven selection enhances stratification accuracy and clinical applicability in OSAS.

CRP, SII, and Fibrinogen are promising and potentially useful biomarkers for evaluating OSAS severity and monitoring treatment response. Integration with machine learning models may support personalized stratification and clinical follow-up.

### 4.6. Clinical Implications

These findings highlight practical, low-cost biomarkers—particularly CRP and SII—that may help clinicians prioritize patients for polysomnography, monitor treatment response, or guide follow-up when repeat PSG is unavailable. Fibrinogen may serve as a complementary marker for tracking systemic recovery after therapy.

### 4.7. Strengths and Limitations

This study offers novel contributions to the understanding of OSAS, particularly in the use of CRP, SII, and Fibrinogen as diagnostic and treatment-responsive biomarkers. CRP demonstrated the highest diagnostic accuracy for severe OSAS (AUC = 0.91), while SII proved to be dynamic and responsive to treatment, with a 21.5% reduction post-therapy. Fibrinogen showed significant changes post-treatment, reinforcing its role as a sensitive marker of systemic inflammation. Additionally, the study’s use of unsupervised clustering identified three distinct inflammatory OSAS subgroups, offering the potential for personalized treatment. Machine learning models validated CRP, SII, and Fibrinogen as top predictors of disease severity, integrating these biomarkers into a predictive analytics framework.

However, the study has limitations. Conducted at a single center, its findings may not be fully generalizable—short-term follow-up limits understanding of long-term biomarker trajectories. Potential confounding factors, such as medication or diet, were not controlled, and although anatomical and polysomnographic criteria guided treatment, residual confounding by indication and incomplete CPAP adherence monitoring may have influenced biomarker changes. We did not apply propensity score methods or inverse probability weighting to mitigate confounding by indication; future studies should address this before comparing CPAP and surgical outcomes. In addition, the wide inclusion age range (18–70 years) introduces a potential age-related inflammatory bias (‘inflammaging’). Older adults may exhibit chronically elevated CRP and Fibrinogen independent of OSAS, which could inflate baseline biomarker values and partially confound severity associations despite adjustment for BMI, sex, and PSG stage

## 5. Future Directions

Future research should aim to identify novel OSAS-specific inflammatory and metabolic markers with higher sensitivity and disease-stage correlation, potentially through multi-omics approaches. Long-term follow-up across different age groups, sexes, and comorbidity profiles will clarify biomarker stability and support risk stratification in personalized management. The machine learning findings from this study should be tested in external cohorts. Integrating biomarker-based models into clinical decision tools could improve early diagnosis and individualized therapy planning. Expanding the use of artificial intelligence for biomarker selection and outcome prediction may accelerate the development of interpretable, scalable, and clinically meaningful algorithms in OSAS care.

## 6. Conclusions

This study demonstrates that CRP, SII, and Fibrinogen are valuable hematologic biomarkers for assessing OSAS severity and treatment response. CRP showed the highest diagnostic accuracy, while SII and Fibrinogen reflected dynamic inflammatory changes. Cluster analysis revealed distinct inflammatory subtypes, and machine learning enhanced biomarker selection. These findings support the use of routine blood markers, combined with AI tools, for cost-effective OSAS diagnosis and personalized care. Further studies should validate these models and explore more specific biomarkers.

## Figures and Tables

**Table 1:** Pre- and Post-Treatment Laboratory and Clinical Parameter Changes with Effect Sizes in OSAS Patients.

Parameter	Female (Mean ± SD)	Male (Mean ± SD)	M-F Diff	M-F %	Significance		Pre (All)	Post (All)	Pre-Post Diff	Pre-Post %	Significance Pre-Post *p*	*p*-Value	Wilcoxon Stat	Cohen’s d
					M-F *t*-Test	M-F *p* Value								
**Neck** **Circuference**	40.04 ± 3.726	40.29 ± 3.657	0.2559	0.6392	0.484	0.6289	40.17 ± 3.684	39.57 ± 3.689	0.5945	1.48	*p* < 0.0001	0.000	0.00	2.07
**BMI**	29.26 ± 4.976	30.4 ± 5.262	1.138	3.89	1.552	0.1222	29.83 ± 5.14	29.06 ± 5.145	0.7735	2.593	*p* < 0.0001	0.000	0.00	1.89
**PSG Stage**	2.546 ± 1.109	2.51 ± 1.028	−0.03619	−1.421	−0.2363	0.8135	2.528 ± 1.066	1.713 ± 0.7248	0.8154	32.25	*p* < 0.0001	0.000	0.00	1.24
**OSAS Severity**	1.495 ± 0.5026	1.561 ± 0.4988	0.06638	4.441	0.9256	0.3558	1.528 ± 0.5005	1.159 ± 0.3666	0.3692	24.16	*p* < 0.0001	0.000	0.00	0.76
**CRP**	6.203 ± 1.667	6.287 ± 1.503	0.08415	1.357	0.3701	0.7117	6.245 ± 1.583	5.38 ± 1.462	0.8653	13.86	*p* < 0.0001	0.000	0.00	2.55
**ESR**	18.9 ± 5.129	19.2 ± 4.933	0.3019	1.598	0.4189	0.6757	19.05 ± 5.021	16.43 ± 4.712	2.627	13.79	*p* < 0.0001	0.000	0.00	2.70
**Fibrinogen**	497.9 ± 103	515.6 ± 103.4	17.73	3.562	1.2	0.2316	506.8 ± 103.3	436.3 ± 99.14	70.53	13.92	*p* < 0.0001	0.000	0.00	2.83
**Neutrophils**	6.215 ± 1.758	6.391 ± 1.475	0.176	2.831	0.757	0.45	6.303 ± 1.62	5.427 ± 1.495	0.8766	13.91	*p* < 0.0001	0.000	0.00	2.54
**Lymphocytes**	2.469 ± 0.7415	2.529 ± 0.7561	0.05991	2.426	0.5586	0.5771	2.499 ± 0.7475	2.221 ± 0.325	0.278	11.2	*p* < 0.0001	0.000	0,00	32.11
**Monocytes**	0.7582 ± 0.2368	0.7756 ± 0.266	0.01736	2.29	0.4816	0.6306	0.767 ± 0.2514	0.6614 ± 0.2269	0.1056	13.77	*p* < 0.0001	0.000	0.00	2.24
**Platelets**	312.2 ± 68.74	322.1 ± 67.1	9.847	3.154	1.012	0.3128	317.2 ± 67.93	273.1 ± 64.54	44.12	13.91	*p* < 0.0001	0.000	0.00	2.77
**RDW**	13.61 ± 0.5037	13.59 ± 0.5409	−0.01932	−0.1419	−0.2582	0.7965	13.6 ± 0.5214	11.67 ± 0.8437	1.93	14.19	*p* < 0.0001	0.000	0.00	2.88
**NLR**	2.663 ± 0.9797	2.775 ± 1.268	0.1124	4.222	0.693	0.4892	2.719 ± 1.133	2.328 ± 0.9552	0.3915	14.4	*p* < 0.0001	0.000	0.00	1.64
**PLR**	135.7 ± 48.81	138.4 ± 53.87	2.779	2.049	0.3776	0.7061	137 ± 51.3	117.4 ± 44.22	19.67	14.35	*p* < 0.0001	0.000	0.00	1.86
**SII**	826.6 ± 301.6	873.2 ± 330.5	46.55	5.631	1.028	0.3054	850 ± 316.5	629.7 ± 248.2	220.4	25.92	*p* < 0.0001	0.000	0.00	2.00
**MLR**	0.3331 ± 0.1562	0.3326 ± 0.1729	−0.0005418	−0.1626	−0.02297	0.9817	0.3328 ± 0.1643	0.2853 ± 0.1428	0.04754	14.28	*p* < 0.0001	0.000	0.00	1.59
**PIV**	642.2 ± 335.7	692.8 ± 358.7	50.59	7.878	1.017	0.3105	667.6 ± 347.5	433.5 ± 248.7	234.1	35.07	*p* < 0.0001	0.000	0.00	1.73

Note: Data are presented as mean ± SD for normally distributed variables and median (IQR) for non-normal variables. *p*-values from Wilcoxon signed-rank tests were adjusted for multiple comparisons using the Benjamini–Hochberg procedure. Cohen’s d was calculated to assess effect sizes. Significant improvements were observed in PSG Stage, CRP, ESR, and several CBC-derived indices. *n* values vary slightly by biomarker because of missing laboratory draws at follow-up (range 186–195 baseline, 180–195 post-treatment.

## Data Availability

The datasets used and/or analyzed during the current study are available from the corresponding author on reasonable request.
